# Actinotignum schaalii Caught for the Second Time in Fournier's Gangrene!

**DOI:** 10.7759/cureus.13288

**Published:** 2021-02-11

**Authors:** Syeda Sahra, Abdullah Jahangir, Harika Kandlakunta, Allison Glaser

**Affiliations:** 1 Internal Medicine, Northwell Health, Staten Island, USA; 2 Infectious Diseases, Northwell Health, Staten Island, USA

**Keywords:** actinotignum schaali, actinobaculum schaali, fournier’s gangrene

## Abstract

*Actinotignum schaalii, *a Gram-positive rod residing in the urinary tract, is responsible for urinary tract infections and their complications including but not limited to bacteremia and sepsis. *A. schaalii *is increasingly being detected in body fluid specimens owing to advancements in PCR techniques. This report describes an interesting case of an adult diabetic patient managed for Fournier's gangrene. *A. schaalii *was detected in the PCR of his wound cultures. To the best of our knowledge, this is the second case of *A. schaalii* as the causative agent for Fournier's gangrene.

## Introduction

*Actinotignum schaalii,* formerly *Actinobaculum schaalii,* causes urinary tract infections in elderly patients [1-3], and rarely in juvenile patients with pelvi-ureteral junction (PUJ) abnormality [4]. It is reported to cause bacteremia, cellulitis, vertebral osteomyelitis [5], necrotizing fasciitis, and infectious endocarditis [6].

Fournier’s gangrene is necrotizing fasciitis affecting the genital and perineal region predominantly in males [7,8], with a history of diabetes mellitus, alcohol dependency, immobilization, active smoking, and immunosuppression [9,10]. So far, only one case has been reported where it grew in tissue samples taken from Fournier’s gangrene [11]. The pathogen responded appropriately to antibiotics and the patient subsequently recovered from the infection. We present this case to the medical community to be aware of the increasing prevalence and detection of *A. schaalii *in culture studies from body fluids. Culturing of* A. schaalii* has been historically difficult. With the advent of modified techniques in detecting molecular targets in PCRs with high accuracy, the incidence of *A. schaalii* is expected to be higher in the future.

## Case presentation

A 45-year-old gentleman, an active smoker, with a prior medical history of morbid obesity, diabetes (controlled, latest hemoglobin a1c: 5.6 mmol/mol), sleep apnoea, hypothyroidism, narcolepsy, and chronic back pain with a history of L3-L4 laminectomy presented to the emergency room with testicular pain for past one day. His pain started the night before presentation when he noticed a painful boil on his right upper thigh, which progressively got worse over the day. The boil was associated with subjective fevers and chills.

The vital signs and blood work on presentation were suggestive of sepsis (temperature: 101.7 F, HR 131 beats per minute, respiratory rate of 22 breaths per minute, WBC 22.6 with predominant neutrophilia, lactate 1.9 mmol/L). On physical examination, the right inguinal region was erythematous, tender to palpation, and indurated with deep crepitus. The scrotum overall was enlarged, with the right hemi-scrotum being erythematous without induration or crepitus.

**Figure 1:**
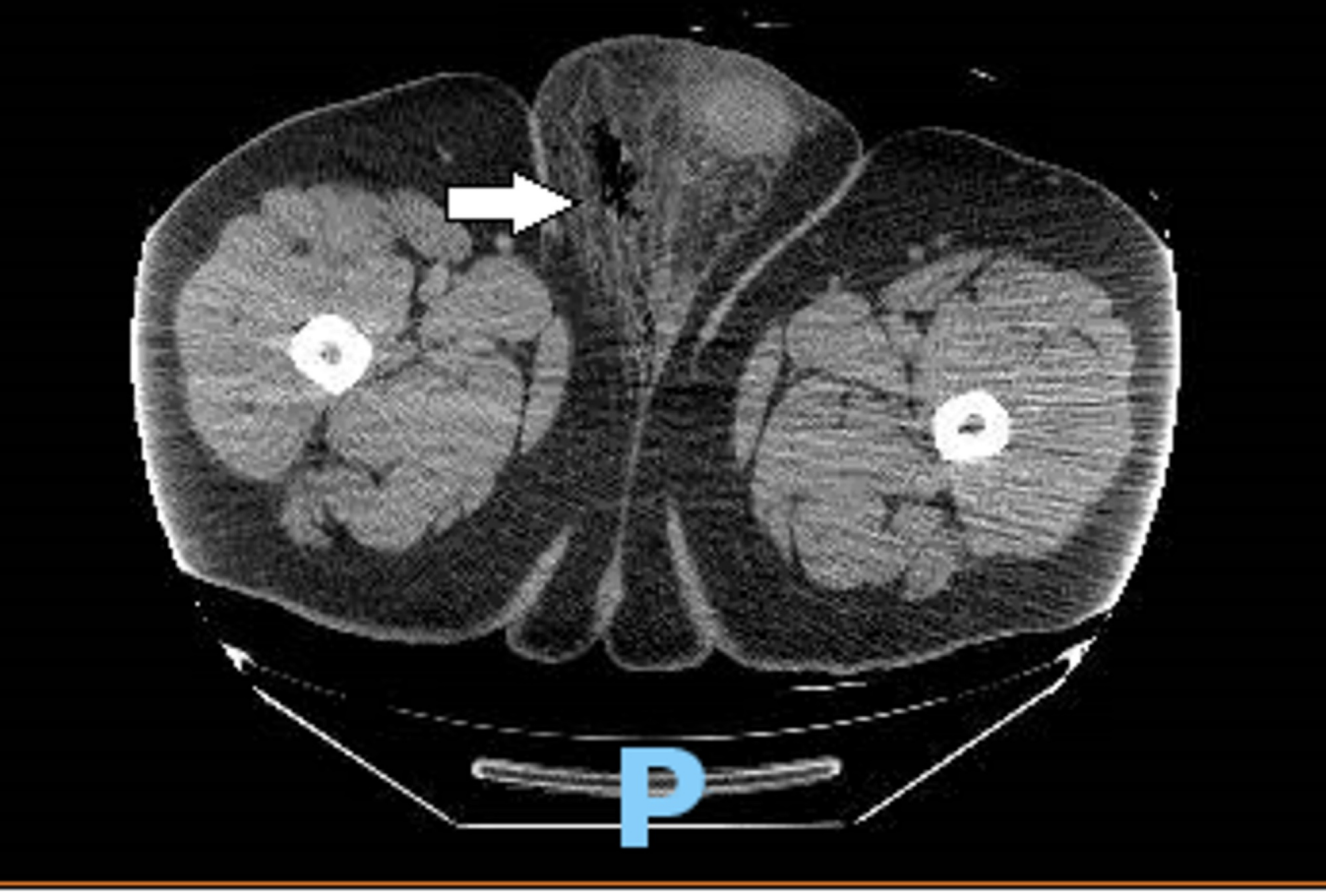
Computed tomography of abdomen and pelvis showing inflammatory changes and gas noted in the right inguinal region with extension into the right side of the scrotum and scrotal edema.

CT of abdomen and pelvis was performed, which showed inflammatory changes and gas (consistent with infection with the gas-forming organism, e.g., Fournier's gangrene) noted in the right inguinal region with extension into the right side of the scrotum and scrotal edema. No extension of inflammation into the pelvis itself or perianal/perirectal region was seen (Figure 1).

The patient was started on intravenous fluids and antibiotics and underwent incision and debridement of right scrotum soft tissue on hospital day 2. Operative findings were significant for right groin necrotic tissue, deep to subcutaneous tissue, and fascia. The necrotic tissue was debrided, tissue samples were sent for Gram staining and cultures. Blood cultures were negative. Initial wound culture and sensitivity showed moderate anaerobic Gram-negative Rods. Vancomycin was stopped and IV cefepime and IV clindamycin were continued.

Repeat debridement of the right scrotum was done three days later. Operative findings showed granulating wounds with purulent drainage and fat necrosis. The cultures from the first debridement showed* A. schaalii* group detected on Bruker MALDI Biotyper (MALDI-TOF MS system, Bruker Daltonics, Billerica, MA). Antibiotics were switched to Rocephin and clindamycin.

He was discharged home on oral Augmentin for eight more days. The patient was re-evaluated four months later. The site of the previous infection in the right hemiscrotum was healing appropriately.

## Discussion

*Actinotignum schaalii, Actinotignum urinale, and Actinotignum sanguinis* are included in the genus Actinotignum [12]. *A. schaalii* (reclassified in 1997 [13]) is one of the slow-growing, Gram-positive, catalase-negative, facultative rods that inhabit the urinary tract [12]. Multiple pathogens are responsible for necrotizing fasciitis of the groin called Fournier’s gangrene. The mortality rate is exceptionally high in high-risk individuals (with diabetes mellitus, underlying immunosuppression, and tobacco abuse).

Our patient was predisposed to Fournier's gangrene as he was obese, an active smoker, and diabetic. Like mentioned earlier, *A. schaalii* is present in urinary tracts. The immunosuppression from smoking and diabetes can lead to the exponential growth of* A. schaalii*, leading to necrotizing fasciitis in the groin area. Fournier's gangrene is traditionally only reported in males. The pathogens most commonly encountered in Fournier's gangrene include g*roup A Streptococcus, Staphylococcus aureus, Pseudomonas, *and *E. Coli*. To the best of our knowledge, this is the second case of the presence of *A. schaalii *being documented in Fournier’s gangrene [11]. There is a possibility of genital and perianal gangrene and necrotizing fasciitis due to *A. schaalii *in the female population as well as risk factors like diabetes, smoking, alcoholism, and obesity. The invasive nature of *A. schaalii* is evident from its infections in vertebral bodies and septicaemia. The course of treatment can be prolonged with possible recurrence in the presence of co-morbidities like diabetes and active tobacco usage.

## Conclusions

The opportunistic growth of *A. schaalii *from the urinary tract has the potential to cause necrotizing fasciitis. The second case of Fournier's gangrene secondary to *A. schaalii *is being reported. Physicians need to be mindful of its presence in the human urinary tract and increased detection owing to PCR assays.

## References

[1] Bank S, Jensen A, Hansen TM, Søby KM, Prag J (2010). Actinobaculum schaalii, a common uropathogen in elderly patients, Denmark. Emerg Infect Dis.

[2] Sturm PD, Van Eijk J, Veltman S, Meuleman E, Schülin T (2006). Urosepsis with Actinobaculum schaalii and Aerococcus urinae. J Clin Microbiol.

[3] Nielsen HL, Søby KM, Christensen JJ, Prag J (2010). Actinobaculum schaalii: a common cause of urinary tract infection in the elderly population. Bacteriological and clinical characteristics. Scand J Infect Dis.

[4] Pajkrt D, Simoons-Smit AM, Savelkoul PH, van den Hoek (2003). Pyelonephritis caused by Actinobaculum schaalii in a child with pyeloureteral junction obstruction. Eur J Clin Microbiol Infect Dis.

[5] Haller P, Bruderer T, Schaeren S (2007). Vertebral osteomyelitis caused by Actinobaculum schaalii: a difficult-to-diagnose and potentially invasive uropathogen. Eur J Clin Microbiol Infect Dis.

[6] Hoenigl M, Leitner E, Valentin T, Zarfel G, Salzer HJ, Krause R, Grisold AJ (2010). Endocarditis caused by Actinobaculum schaalii, Austria. Emerg Infect Dis.

[7] Eke N (2000). Fournier's gangrene: a review of 1726 cases. Br J Surg.

[8] Sorensen MD, Krieger JN, Rivara FP, Broghammer JA, Klein MB, Mack CD, Wessells H (2009). Fournier's Gangrene: population based epidemiology and outcomes. J Urol.

[9] Czymek R, Hildebrand P, Kleemann M (2009). New insights into the epidemiology and etiology of Fournier's gangrene: a review of 33 patients. Infection.

[10] Ghadian A (2012). Actinobaculum schaalii as a uropathogen in immunocompromised hosts. Iran J Kidney Dis.

[11] Vanden Bempt I, Van Trappen S, Cleenwerck I, De Vos P, Camps K, Celens A, Van De Vyvere M (2011). Actinobaculum schaalii causing Fournier's gangrene. J Clin Microbiol.

[12] Lotte R, Lotte L, Ruimy R (2016). Actinotignum schaalii (formerly Actinobaculum schaalii): a newly recognized pathogen-review of the literature. Clin Microbiol Infect.

[13] Lawson PA, Falsen E, Akervall E, Vandamme P, Collins MD (1997). Characterization of some Actinomyces-like isolates from human clinical specimens: reclassification of Actinomyces suis (Soltys and Spratling) as Actinobaculum suis comb. nov. and description of Actinobaculum schaalii sp. Int J Syst Bacteriol.

